# Fish oil enhanced the efficacy of low-dose cyclophosphamide regimen for proliferative lupus nephritis: a randomized controlled double-blind trial

**DOI:** 10.29219/fnr.v65.7842

**Published:** 2021-07-26

**Authors:** Chi Zhang, Chang Ge, Junsheng Wang, Dong Sun

**Affiliations:** 1 Department of Nephrology, The Affiliated Suqian Hospital of Xuzhou Medical University, Jiangsu, China; 2 Xuzhou Medical University, Jiangsu, China; 3 Department of Otorhinolaryngology-Head and Neck Surgery, The Affiliated Suqian Hospital of Xuzhou Medical University, Jiangsu, China; 4 Department of Nephrology, The Affiliated Hospital of Xuzhou Medical University, Jiangsu, China; 5 Department of Internal Medicine and Diagnostics, Xuzhou Medical University, Jiangsu, China

**Keywords:** lupus nephritis, systemic lupus erythematosus, fish oil, cyclophosphamide, treatment, adjuvant

## Abstract

**Background:**

Lupus nephritis (LN) is one of the most severe organ that damages the systemic lupus erythematosus (SLE). Cyclophosphamide is one of the main drugs used in the treatment of LN. Fish oil is a general term of all the oily substances in fish, whose main component is omega-3 fatty acid. This study aimed to investigate whether fish oil could be used as an adjunct to low-dose cyclophosphamide in proliferative LN treatment.

**Methods:**

A total of 237 patients with proliferative LN were recruited and randomized into two groups: cyclophosphamide + placebo group and cyclophosphamide + fish oil group. In the cyclophosphamide + placebo group, participants received prednisone + cyclophosphamide + placebo. In the cyclophosphamide + fish oil group, participants received prednisone + cyclophosphamide + fish oil. Before and after treatment, the clinical parameters of the patients in both groups were evaluated.

**Results:**

In the cyclophosphamide + fish oil group, the number of patients achieving complete remission (*n* = 45, 46.9%) was significantly higher than the cyclophosphamide + placebo group (*n* = 31, 32.6%). The number of patients achieving no response in the cyclophosphamide + fish oil group (*n* = 8, 8.3%) was significantly lower than the cyclophosphamide + placebo group (*n* = 22, 23.2%). Hematuria (*P* = 0.036), urine protein-creatinine ratio (uPCR) (*P* = 0.014), estimated glomerular filtration rate (eGFR) (*P* = 0.027), and renal SLE disease activity index (SLEDAI) (*P* = 0.009) improved more significantly in the cyclophosphamide + fish oil group. The number of patients with infection (*P* = 0.04) or urinary tract infection (*P* = 0.04) in the cyclophosphamide + fish oil group was lower than the cyclophosphamide + placebo group.

**Conclusion:**

In conclusion, the treatment of fish oil in LN patients enhances the efficiency of cyclophosphamide, alleviates nephritis-related parameters, and inhibits infection and urinary tract infection during the treatment. Thus, fish oil may serve as a potential adjuvant drug in the treatment of LN.

## Popular scientific summary

Lupus nephritis can seriouly damage the health. We try to answer the question whether fish oil could be used as an adjunct to low-dose cyclophosphamide for the treatment of lupus nephritis. Our results are promising, as the treatment of fish oil in the patients enhanced the efficiency of cyclophosphamide, alleviated nephritis-related impairments. We conclude that fish oil may serve as a potential adjuvant drug in the treatment of lupus nephritis.

Systemic lupus erythematosus (SLE) is an autoimmune disease, mainly due to the loss of immune tolerance against endogenous nucleic acid, resulting in systemic autoimmunity and organ damages (1). Lupus nephritis (LN) is a type of severe organ damage in SLE (2). Over the past few decades, the genetic and pathogenic mechanisms of LN have been investigated. However, despite a better understanding of LN and progressive improvements in clinical treatment strategies have been achieved, LN is still one of the main causes of mortality in SLE patients (3). Cyclophosphamide is one of the main drugs used in the treatment of LN (3). Several pivotal clinical trials have demonstrated the effectiveness of cyclophosphamide in the treatment of proliferative LN (4, 5).

Fish oil is a general term of all oily substances in fish, derived from the bodies of large marine fish, whose main component is polyunsaturated omega-3 fatty acid (6). Fish oil reduces inflammatory reactions, lowers blood lipids, and prevents cardiovascular diseases (7). Fish oil has important clinical applications in the treatment of inflammatory diseases, especially rheumatoid diseases (8, 9). Omega-3 fatty acid achieves its therapeutic effect mainly by inhibiting the inflammatory response (10). Results from animal and clinical trials showed that fish oil had an inhibitory effect on LN (11–13). The therapeutic efficacy of fish oil on LN is not satisfactory, the inhibitory effect on LN remains weaker than that of standard therapeutic drugs, and longer-term clinical trials demonstrating their effectiveness are lacking. However, fish oil can be an ideal complementary drug because of its low price, better safety profile, fewer side effects, and many health benefits. On the other hand, the guiding therapeutic drugs for LN such as cyclophosphamide have more serious side effects in high doses and have unsatisfactory efficacy in low doses (14). Therefore, cyclophosphamide with a complementary drug without significant side effects for clinical treatment is a very reasonable treatment plan.

In this intent-to-treat clinical study, fish oil was used as an adjunct to low-dose cyclophosphamide to assess the potency of fish oil on the efficacy of cyclophosphamide in proliferative LN therapy.

## Methods

### Patient eligibility

In this randomized controlled trial, all patients with proliferative LN were enrolled consecutively in the Affiliated Suqian Hospital of Xuzhou Medical University. This research was approved by the Ethics Committee of the Affiliated Suqian Hospital of Xuzhou Medical University.

Inclusion criteria: 1) According to the criteria of the American College of Rheumatology, patients were diagnosed as SLE, with an SLE disease activity index (SLEDAI) ≥ 8. 2) Patient age ≥ 18. 3) Patients had 24-h urine protein ≥ 500 mg and/or routine urinalysis through microscopy showed active cellular phenotype/deposition. 4) Based on the International Society of Nephrology/Renal Pathology Society (ISN/RPS), all the patients had biopsy-proved diffuse or focal proliferative LN (Type III/IV A or A/C).

Exclusion criteria: 1) Patients who had received intravenous or oral cyclophosphamide, mycophenolate, cyclosporine, or steroids > 15 mg/day within the last 3 months. 2) Patients with chronic renal failure, renal thrombotic microangiopathy, diabetes mellitus, malignancy, or coronary artery disease. 3) Patients who had previously demonstrated severe toxicity to immunosuppressive drugs. 4) Patients who had acute or chronic infections. 5) Patients who were pregnant.

### Interventions

**Fig. 1:**
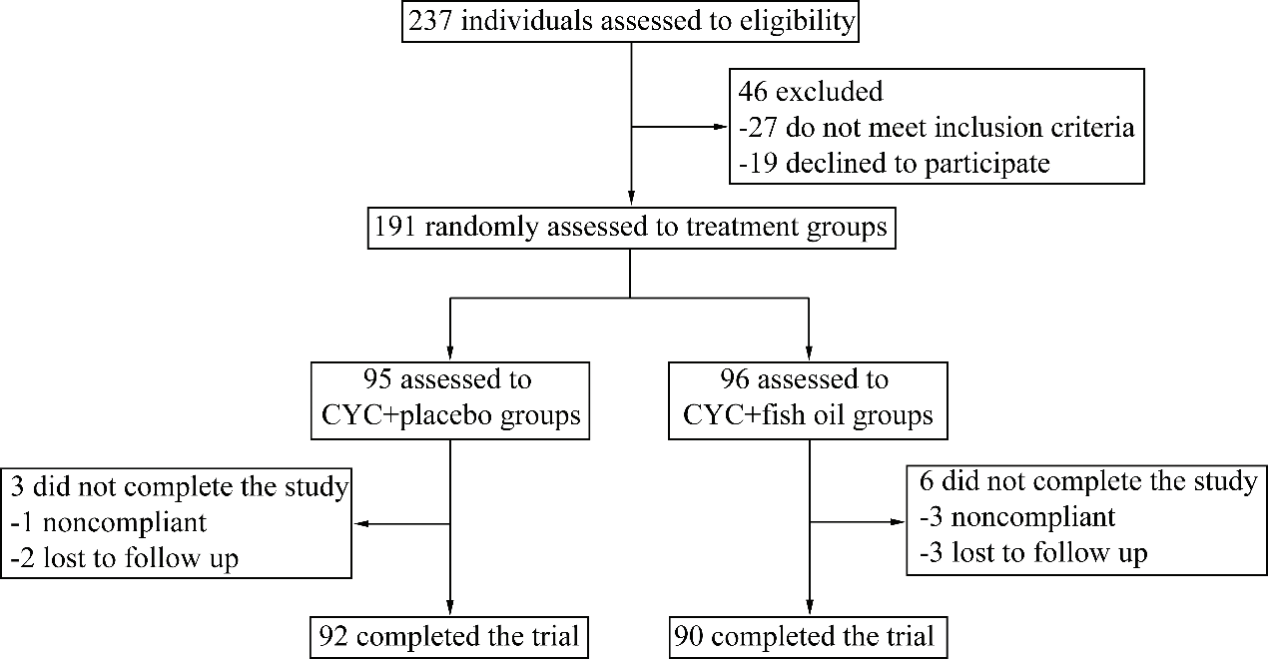
Disposition of the study participants enrolled and follow-up.

A total of 237 LN patients were recruited, and 46 of them were excluded. The 191 eligible participants were randomized into 2 groups: cyclophosphamide + placebo group (*n* = 95) and cyclophosphamide + fish oil group (*n* = 96). Ninety-two participants in the cyclophosphamide + placebo group and 90 participants in the cyclophosphamide + fish oil group completed the trial. Nine patients did not complete this study (three in the control group and six in the fish oil group), and they were deemed as treatment failure in the intention-to-treat analysis. The research framework of this study was shown in Fig. 1. Demographic data and baseline characteristics of participants were recorded. Researchers involved in the recruitment, data collection, and analyses were blind to group assignment.

In the cyclophosphamide + placebo group, participants received prednisone + cyclophosphamide + placebo. In the cyclophosphamide + fish oil group, participants received prednisone + cyclophosphamide + fish oil. All patients received prednisone (1.0 mg/kg/day) for 8 weeks. Dosage of prednisone was then tapered by 5–10 mg every 2 weeks until it was reduced to 20 mg/day, and then tapered by 2.5 mg every 2 weeks until a dose of 10 mg/day was reached and maintained to the endpoint of the trial. The maximum dose of prednisone did not exceed 60 mg/day. All the patients received a 6-month cyclic treatment with low-dose cyclophosphamide, administered intravenously at a dose of 0.5 g/month. The dosages of prednisone and cyclophosphamide were modified from the previous reports (15, 16). Patients received three capsules with placebo or fish oil per day for 8 months. Capsule contained 1 g of fish oil (101 mg docosahexaenoic acid and 78 mg eicosapentaenoic acid) or placebo (contained 1 g of corn germ oil), as reported (17). All white capsules were labeled with a numeric code. Improvement in LN, differences in relevant pathological indicators, and adverse events were assessed after 1 year (52 weeks).

### Clinical parameters

Various clinical parameters of LN patients were monitored before and after treatment. The clinical parameters included 24-h proteinuria, hematuria, hypertension, serum creatinine, serum albumin, serum complement 3 (C3), serum C4, urine protein–creatinine ratio (uPCR), anti-double-stranded DNA (anti-dsDNA) antibodies, estimated glomerular filtration rate (eGFR), and renal SLEDAI.

The levels of urine protein and creatinine were analyzed through the Hitachi 7180 Autoanalyzer (Hitachi, Tokyo, Japan). uPCR (mg/g) = urine total protein (mg/dL) * 1000/(urine creatinine, mg/dL). 24-h urine protein = 24 h urine volume (mL) * urine protein (mg/dL). The Cockcroft-Gault formula was used for the calculation of eGFR. Radioimmunoassay (EUROIMMUN AG, Germany) was used for the evaluation of serum antibodies against dsDNA.

### Renal responses

At the endpoint of the clinical trial (week 52), renal responses were assessed in both groups. Complete remission was defined as uPCR < 0.5 g, GFR > 90 mL/min or stable renal function (<10% decrease relative to baseline if previous GFR was abnormal), and inactive urinary sediments.

Partial remission was defined as ≥50% reduction of proteinuria to sub-nephrotic levels, GFR > 90 mL/min or stable renal function (<10% decrease relative to baseline if previous GFR was abnormal), and inactive urinary sediments.

Non-response was defined as the criteria for CR or PR were not met, or if the patients experienced severe diseases.

### Statistical analysis

SPSS 22.0 was used for data analysis. Data were expressed as mean ± standard deviation (SD). Means for the two groups were compared using Student’s *t*-test or Mann–Whitney U test. Proportions were compared using Chi-square (χ^2^) test. *P* < 0.05 was thought to indicate statistical significance.

## Results

### Demographic data of participants

The demographic data of these two groups were shown in Table 1. Based on the results of statistical analysis, these two groups were homogenous for gender, age of SLE onset, age of nephritis onset, and disease duration (all *P* > 0 .05). Clinical features at the time of nephritis in these two groups were also shown in Table 1. No statistically differences were found in the clinical features between these two groups, including fever/systemic, mucocutaneous, arthritis, hemolytic anemia, leucopenia, thrombocytopenia, pericardial effusion, pleural effusion, vasculitis, myositis, and myocarditis (all *P*  > 0 .05).

**Table 1:** Demographic data and baseline characteristics of participants

	Cyclophosphamide + placebo (*n* = 95)	Cyclophosphamide + fish oil (*n* = 96)	*P*
Female/male	82/13	84/12	0.81
Age of SLE onset^a^	28.5 ± 10.05	26.7 ± 10.35	0.22^a^
Age of onset of nephritis^a^	30.6 ± 10.01	29.2 ± 10.51	0.35^a^
Disease duration, months (median^b^)	19 (5–37)	16 (6.5–30)	0.38^b^
Clinical features at the time of nephritis^c^	-	-	-
Fever/systemic	15 (15.8)	21 (21.9)	0.28
Mucocutaneous	51 (53.7)	46 (47.9)	0.42
Arthritis	35 (36.8)	29 (30.2)	0.33
Hemolytic anemia	21 (22.1)	30 (31.3)	0.15
Leucopenia	31 (32.6)	26 (27.1)	0.40
Thrombocytopenia	18 (18.9)	19 (19.8)	0.88
Pericardial effusion	4 (4.2)	5 (5.2)	0.74
Pleural effusion	13 (13.7)	15 (15.6)	0.70
Vasculitis	21 (22.1)	17 (17.7)	0.45
Myositis	9 (9.5)	7 (7.3)	0.59
Myocarditis	2 (2.1)	6 (6.3)	0.15

Values expressed as number of patients, number (percentage), or mean ± standard deviation or mean (interquartile range). All patients had proliferative lupus nephritis.

a Student’s *t* test.

b Compared using non-parametric Mann–Whitney U test.

c χ^2^ test.

### Change of parameters in patients after treatment

In this study, all patients were evaluated for nephritis-related parameters before treatment. The results showed no significant differences between groups in terms of white blood cells (WBCs), proteinuria, hematuria, hypertension, serum albumin, serum creatinine, uPCR, eGFR, low complements, anti-dsDNA, renal SLEDAI, and pathologic type (all *P* > 0 .05) (Table 2).

**Table 2:** Baseline renal parameters

	Cyclophosphamide + placebo (*n* = 95)	Cyclophosphamide + fish oil (*n* = 96)	*P*-value
WBC (×10^9^/L)	9.1 ± 3.8	8.6 ± 4.1	0.38
Proteinuria (g/24 h)^a^	4.1 (1.3–5.9)	3.8 (1.2–6.1)	0.54
Hematuria	91 (95.8)	93 (96.9)	0.69
Hypertension*	45 (47.4)	53 (55.2)	0.28
Serum albumin (g/dL)^a,b^	2.40 ± 0.52	2.40 ± 0.61	0.22
Serum creatinine (g/dL)^a,c^	1.12 (0.5–3.5)	0.94 (0.6–3.6)	0.23
uPCR (gm/day)	1.5 ± 0.85	1.4 ± 0.92	0.44
eGFR (mL/min/1.73 m^2^)	67.86 ± 25.8	68.14 ± 24.1	0.94
Low complements^#^	73 (76.8)	71 (74)	0.64
Anti-dsDNA^$^	77 (81.1)	71 (74)	0.24
Renal SLEDAI	17.6 ± 5.8	18.6 ± 7.4	0.3
Pathologic type	-	-	0.9
Class III	5 (5.3)	3 (3.1)	-
Class IV	67 (70.5)	69 (71.9)	-
Class V	9 (9.5)	9 (9.4)	-
Class V+IV or V+III	14 (14.7)	15 (15.6)	-

Values expressed as number of patients, number (percentage), or mean ± standard deviation or mean (interquartile range).

χ^2^ test, Mann–Whitney *U* test, or Student’s *t* test was used.

* Diastolic BP > 90 mmHg.

# Low C3 < 0.90 g/L and low C4 < 0.10 g/L

$ Anti-dsDNA >60 IU/mL

a Reference range for serum albumin is 3.5–5.0 g/dL.

b Reference range for serum creatinine is 0.59–1.47 mg/dL.

After a 52-week treatment, the efficacy of treatment in both groups was evaluated. In the cyclophosphamide + fish oil group, patients achieving complete remission (*n* = 45, 46.9%) were dramatically higher than the cyclophosphamide + placebo group (*n* = 31, 32.6%) (*P* = 0.04) (Table 3). As depicted in Table 3, no difference between the cyclophosphamide + fish oil group (*n* = 43, 44.8%) and cyclophosphamide + placebo group (*n* = 42, 44.2%) was observed with respect to partial remission (*P* = 0.94). However, the number of the patients achieving no response in the cyclophosphamide + fish oil group (*n* = 8, 8.3%) was dramatically lower than the cyclophosphamide + placebo group (*n* = 22, 23.2%) (*P* = 0.005) (Table 3).

**Table 3:** Efficacy of treatment in both groups

	Cyclophosphamide + placebo (*n* = 95), *n* (%)	Cyclophosphamide + fish oil (*n* = 96), *n* (%)	P*	
Complete remission	31 (32.6)	45 (46.9)	0.04	0.01
Partial remission	42 (44.2)	43 (44.8)	0.94	
No response	22 (23.2)	8 (8.3)	0.005	

* χ^2^ test was used.

Nephritis-related parameters were also evaluated in both groups after treatment. As shown in Table 4, in both groups, 24-h proteinuria, serum creatinine, uPCR, anti-dsDNA antibody concentration, renal SLEDAI, and the numbers of patients with hematuria, hypertension, and low complements were all decreased following the 52-week treatment. However, serum albumin and eGFR were increased after treatment (Table 4).

**Table 4:** Comparison of various parameters in patients before and after treatment in both groups

	Cyclophosphamide + placebo (*n* = 95)		Cyclophosphamide + fish oil (*n* = 96)		*P*-value (after treatment)
	Before treatment	After treatment	Before treatment	After treatment	
Proteinuria (g/24 h)	4.1 (1.3–5.9)	1.5 (0.6–5.4)	3.8 (1.2–6.1)	1.8 (0.5–5.1)	0.68
Hematuria	91 (95.8)	54 (56.8)	93 (96.9)	40 (42.1)	0.036
Hypertension*	45 (47.4)	37 (38.9)	53 (55.2)	34 (35.8)	0.61
Serum albumin (g/dL)^a^	2.40 ± 0.52	3.70 ± 0.72	2.40 ± 0.61	3.90 ± 0.81	0.07
Serum creatinine (g/dL)^b^	1.12 (0.5–3.5)	0.98 (0.5–3.7)	0.94 (0.6–3.6)	0.91 (0.5–3.4)	0.81
uPCR (gm/day)	1.5 ± 0.85	0.41 ± 0.25	1.4 ± 0.92	0.33 ± 0.19	0.014
eGFR (mL/min/1.73 m^2^)	67.86 ± 25.8	77.91 ± 24.6	68.14 ± 24.1	85.28 ± 21.1	0.027
Low complements^#^	73 (76.8)	35 (36.8)	71 (74)	24 (25.3)	0.076
Anti-dsDNA^$^	77 (81.1)	41 (43.2)	71 (74)	31 (32.6)	0.12
Renal SLEDAI	17.6 ± 5.8	9.29 ± 2.5	18.6 ± 7.4	8.36 ± 2.4	0.009

Values expressed as number of patients, number (percentage), or mean ± standard deviation or mean (interquartile range). χ^2^ test, Mann–Whitney *U*-test, or Student’s *t*-test was used.

* Diastolic BP >90 mmHg.

# Low C3 < 0.90 g/L and low C4< 0.10 g/L.

$ Anti-dsDNA >60 IU/mL.

a Reference range for serum albumin is 3.5–5.0 g/dL.

b Reference range for serum creatinine is 0.59–1.47 mg/dL.

The differences in the nephritis-related parameters between the groups after treatment were also listed in Table 4. The four parameters, hematuria (*P* = 0.036), uPCR (*P* = 0.014), eGFR (*P* = 0.027), and renal SLEDAI (*P* = 0.009), were improved more significantly in the cyclophosphamide + fish oil group.

### Adverse events during the treatment

In this research, serious adverse events reported by >2% of patients in any group were recorded. As shown in Table 5, there was no significant difference in the rate of most of adverse events between the two groups (all *P*  > 0 .05). These adverse events included upper respiratory infection, pneumonia, skin tissue infections, diarrhea, nausea, headache, leukopenia, and alanine transaminase/aspartate transaminase (ALT/AST) rise. Meanwhile, in the cyclophosphamide + fish oil group, patients with infection (*P* = 0.04) or urinary tract infection (*P* = 0.04) were dramatically low than the cyclophosphamide + placebo group (Table 5).

**Table 5:** Serious adverse events reported by >2% of patients in any treatment group

	Cyclophosphamide + placebo (*n* = 95), *n* (%)	Cyclophosphamide + fish oil (*n* = 96), *n* (%)	P*
Infection	15 (15.8)	6 (6.2)	0.04
Upper respiratory infection	4 (4.2)	2 (2.1)	n.s.
Pneumonia	2 (2.1)	3 (3.1)	n.s.
Urinary tract infection	10 (10.5)	3 (3.1)	0.04
Skin tissue infections	4 (4.2)	3 (3.1)	n.s.
Diarrhea	2 (2.1)	2 (2.1)	n.s.
Nausea	2 (2.1)	0 (0.0)	n.s.
Headache	2 (2.1)	1 (1.0)	n.s.
Leukopenia^$^	1 (1.1)	2 (2.1)	n.s.
ALT/AST rise^#^	3 (3.2)	1 (1.0)	n.s.

$ Leukopenia was defined as a white blood cell count below 3 × 10^9^/L.

# ALT/AST, alanine transaminase/aspartate transaminase. An ALT/AST rise was defined as an alanine transaminase or aspartate transaminase level above 1.5 × ULN (the upper limit of the normal range).

* χ^2^ test was used. n.s., not significant.

## Discussion

SLE has been gaining attention in recent years. SLE causes multi-system damage, mainly involving the heart, brain, kidneys, and other important organs of the body (18). The data released by Chinese SLE treatment and research group (CSTAR) showed that the global average prevalence of SLE was 12–39/100,000, and the prevalence of SLE in China was 30–70/100,000, which ranked second in the world (19). SLE patients have a 50% chance of developing LN, and the degree of kidney involvement directly affects the prognosis of SLE (20). So, it is important to understand the pathogenesis, related markers, and the treatment of LN.

Since patients with LN require long-term treatment, it is important not only to demonstrate the effectiveness but also to minimize the toxicity of the drugs (21). The main immunosuppressive drugs for LN therapy include mescaline, azathioprine, and cyclophosphamide (22). However, many patients experience adverse drug reactions including infections, leukopenia, and liver damage during the use of related drugs (23). Therefore, there is a need to find drugs with a good safety profile or to reduce the toxicity of related drugs.

Omega-3 polyunsaturated fatty acids are active constituents of fish oil (24). Both animal and clinical studies have confirmed that fish oil can interfere with the progression of renal lesions. Dietary supplementation with fish oil for 20 months significantly reduced renal damage, resulting in reduced proteinuria, no reduction in GFR, and no end-stage uremia in patients with chronic kidney disease (25, 26). Fish oil supplementation minimized the mean changes in serum creatinine and abnormal histopathologic lesions in rats with chronic renal failure caused by calcineurin inhibitors (27).

In recent decades, several studies explored the influence of fish oil on symptoms and renal function in LN patients. It has been reported that 1-year fish oil dietary supplementation did not reduce disease activity or improve renal function but did influence some lipid parameters in LN patients (6). Another study in LN patients showed that fish oil had no influence on proteinuria, GFR, SLEDAI, or steroid consumption (11). All of these experimental evidences suggest that the application of fish oil as the drug is not ideal for the treatment of LN, and that the inhibitory effect on LN remains weaker than that of standard therapeutic drugs. However, fish oil has still shown significant renal protective effects in many kidney diseases.

Cyclophosphamide remains a reliable and effective drug for the induction phase of LN therapy (28). Glucocorticoid plus cyclophosphamide therapy is the traditional therapeutic strategy for LN and is effective in improving the prognosis of LN. Cyclophosphamide has serious side effects at high doses, while low doses are not as effective. Cyclophosphamide with a complementary drug without significant side effects for clinical treatment is a very reasonable treatment plan. Thus, we explored whether fish oil could be used as an adjunct to low-dose cyclophosphamide in the current clinical trial.

In this study, participants were treated with either prednisone + cyclophosphamide + placebo or prednisone + cyclophosphamide + fish oil. The differences in the pathological indicators related to LN between the two groups at the end of the clinical period were evaluated. The clinical manifestations of LN are commonly discovered by urine examination. Evaluation includes kidney function measurement and urinalysis, generally eGFR and creatinine concentration. The damage on glomerular basement membrane during the pathogenesis of LN causes the extravasation of red blood cells into urine (29). Hematuria is associated with proliferative LN and most commonly found in LN classes III and IV (30). SLEDAI stratifies SLE disease activity. After the treatment, 54 (56.8%) participants treated with placebo had hematuria, while only 40 (42.1%) participants treated with fish oil had hematuria. After the treatment, the uPCR of patients in the fish oil group (0.33 ± 0.19 gm/day) was dramatically lower than in the placebo group (0.41 ± 0.25 gm/day). Meanwhile, eGFR in the fish oil group (85.28 ± 21.1 mL/min/1.73 m^2^) was significantly higher than in the placebo group (77.91 ± 24.6 mL/min/1.73 m^2^). Renal SLEDAI in the fish oil group (8.36 ± 2.4) was significantly lower than in the placebo group (9.29 ± 2.5). The results showed that hematuria, uPCR, eGFR, and renal SLEDAI improved more significantly in the fish oil group, indicating that fish oil as an adjunct drug also had a significant effect on reducing the pathological indices of LN.

Remission was assessed at the end of the clinical period in the placebo and fish oil groups, with a 46.9% complete remission rate in patients treated with fish oil, up from 32.6% in patients treated with placebo. Meanwhile, the rate of no response in the fish oil group (8.3%) was significantly lower than in the placebo group (23.2%). Thus, the treatment of prednisone + cyclophosphamide + fish oil was more effective than the treatment of prednisone + cyclophosphamide + placebo.

This study had some limitations. This was a single center study and needs validation by a larger multicenter cohort. The number of participants was not large enough, and the patients had no biopsy after the treatment.

## Conclusion

In conclusion, the treatment of fish oil in LN patients enhances the efficiency of cyclophosphamide, alleviates nephritis-related parameters, and inhibits infection and urinary tract infection during the treatment. Thus, fish oil may serve as a potential adjuvant drug in the treatment of LN.
